# Clinical Evaluation of the Nose: A Cheap and Effective Tool for the Nasal Fracture Diagnosis

**Published:** 2012-01-23

**Authors:** Joaquín Pérez-Guisado

**Affiliations:** ^a^Service of Plastic, Aesthetic and Reconstructive Surgery, Reina Sofía University Hospital, Av. Menéndez Pidal s/n 14004, Córdoba, Spain

## Abstract

**Objective:** An accurate diagnosis of nasal fracture is dependent on a thorough history and physical examination. The purpose of this investigation was to create a simple method to establish the diagnosis of nasal fracture based only on clinical criteria. **Methods:** A retrospective chart review was carried out of 220 patients suspected of nasal fracture admitted to a hospital specializing in occupational injuries in 2003 and 2004. Sensitivity, specificity, and positive/negative predictive value (PPV/NPV) were calculated for each clinical criterion (8), all the possible combinations of 2 clinical criteria (28) and 3 clinical criteria (56). The following clinical criteria were considered for the analysis: epistaxis, periorbital and/or perinasal ecchymosis, nasal wound or laceration, airway obstruction, nasal inflammation, lateral deviation, irregular nasal dorsum, and acute septal injury. Logisitic regression was used to assess statistical significance. **Results:** For any of the 8 criteria, the average sensitivity and negative predictive values for nasal fracture were very low (<35%). However, specificity and positive predictive values were relatively high (>90%) and increased, respectively, when at least 1 criterion was present (92% and 94%, respectively), when 2 clinical criteria were present (98% and 96%, respectively), and when at least 3 clinical criteria were present (100% for both). **Conclusions:** The presentation of the clinical criteria can be a valuable method for the diagnoses of nasal fracture; nevertheless, when these clinical criteria are absent, the possibility of the nasal fracture cannot be ruled out though the possibility is remote.

The nose is considered the single most prominent aesthetic feature of the face and the fracture of nasal bones is the most common bone injury of the adult face and the third most frequent of all body fractures. It is estimated that 40% of facial trauma cases include fractures of the nasal bones.^1^ In fact, each year in the United States, approximately 50 000 people suffer nasal bone fractures.^2^ Motor vehicle crashes and interpersonal violence are the main causes, and alcohol consumption is often a contributing factor.^3^

Nasal bone fractures are generally considered minor injuries^4^; however, important functional and cosmetic defects have been associated with delayed time to treatment, traumatic edema, preexisting nasal deformity, and occult septal injury.^5^ Accurate diagnosis of nasal fractures is dependent on a thorough history and physical examination.^6^ Patients usually present with some combination of epistaxis, ecchymosis, deformity, tenderness, edema, instability, and crepitation; however, these features may not always be present and are often transient.^7^

Radiography (x-ray) is the standard imaging procedure for suspected nasal fracture. However, its utility for clinical decision making is highly controversial. For detection of fractures of the nasal dorsum, x-ray has high sensitivity (88%) and specificity (95%); nevertheless, for fractures of the lateral nasal wall, specificity is higher (75%) than sensitivity (28%).^8^ Computed Tomography (CT) scans have greater sensitivity and specificity for nasal fracture, but their cost, radiation exposure, and lack of impact on management do not justify their use in diagnosing isolated nasal fractures but when managing the patient with extensive maxillofacial trauma.^9^

For those physicians and specialists with a high degree of experience in treating nasal fractures, an accurate diagnosis is often obtained with only a thorough history and physical examination. Thus, there is potential that the identification of several clinical diagnoses can assist general and emergency physicians in establishing a diagnosis of nasal fracture based simply on clinical history. The value of clinical diagnostic criteria for nasal fractures has not been formally assessed either alone or grouped. The purpose of this investigation was to retrospectively review clinical criteria of fracture to determine an accurate diagnosis of nasal fracture.

## METHODS

### Study design

The study was a retrospective chart review to estimate the predictive value of clinical criteria for predicting diagnosis of nasal fracture. Study information was obtained from a database maintained by the Maxillofacial Surgery Department of “Hospital del Trabajador” (Chile), a hospital specializing in occupational injuries. Since the study was a retrospective chart review and no patient contact was required, authorization from the hospital's ethics committee was not necessary.

### Selection of participants

Records for all patients admitted from January 2003 to December 2004 were reviewed. All patients presenting with nasal trauma and whose medical records included a detailed clinical history were included in the study.

### Methods of measurement

The definitive clinical diagnosis or “gold standard” of nasal fracture (nasal dorsum and nasal wall) is made on the basis of all clinical data combined with x-ray findings (nasal bones and waters projections), intraoperative findings, and CT scans.^8^ Clinical data were compiled through a review of medical history that included the force, direction, and mechanism of injury; the presence of epistaxis or cerebrospinal fluid rhinorrhea at the time of the consultation; any history of previous nasal fracture or surgery, nasal obstruction, and epistaxis or external nasal deformity appreciated by the patient after the injury; signs from the examination of the external nose: periorbital and/or perinasal ecchymosis, nasal wound or laceration, airway obstruction, nasal inflammation, lateral deviation, and irregular nasal dorsum; inspection of the internal nose; and palpation of the nasal bones.

### Data collection and processing

The following 8 clinical criteria were compared with the definitive clinical diagnosis: epistaxis (EPI), periorbital and/or perinasal ecchymosis (ECH), nasal wound or laceration, airway obstruction, nasal inflammation (INF), lateral deviation, irregular nasal dorsum, and acute septal injury (ASI). A positive ASI was a tear, laceration, hematoma, or fracture. If a sign was not listed in the initial report, the finding was assumed to be negative. For the analysis, these findings were categorized as positive or negative.

### Primary data analysis

Ninety-two comparisons with the gold standard were carried out using 1 clinical criterion (8), all the possible combinations of 2 clinical criteria (28 combinations without repetition = C_8,2_) and 3 clinical criteria (56 combinations without repetition = C_8,3_). Sensitivities and specificities were compared using the McNemar test for paired samples with 2 tails. Predictive values were compared using the Fisher exact test with 2 tails. The level of significance was corrected with the Bonferroni test to correct for multiple comparisons.

Decimals resulting from the analysis of the data for the prevalence, sensitivity, and predictive values were not considered and rounding to a whole number was done. The association between nasal fracture and clinical criteria was further examined using multivariate logistic regression. Statistical significance was tested by calculating 95% confidence intervals (CIs) based on an exact binomial distribution. Odds ratios (ORs) and their corresponding 95% CIs were calculated. The analysis was carried out using the SPSS software package, version 17.0 (Chicago, Ill).

## RESULTS

Overall, 220 patients were included in the study (Table 1). Mean age was 36 ± 25 years and 71% were men. A small number (13%) of patients had had previous nasal trauma. The most common injury mechanism was motor vehicle collision (35%).

The prevalence, sensitivity, specificity, and positive and negative predictive values (PPV and NPV) of the 8 clinical criteria are shown in Table 2. Seventy-six percent (N = 167) of the patients had a nasal fracture. The single clinical criterion with the highest sensitivity and NPV was for epistaxis (69% and 50%). When at least 1 clinical criterion was present, sensitivity was 34% and NPV 32%; however, specificity was 92% and PPV 94%. The single clinical criterion with the highest specificity and PPV was ASI (100% and 100%) followed by ecchymosis (98% and 98%), airway obstruction, and lateral deviation (both had identical values of 96% and 96%).

Table 3 presents the prevalence, sensitivity, specificity, PPV, and NPV for each 2-way combination of clinical criteria and for when a combination of 3 clinical criteria are present. The overall sensitivity rate for the detection of nasal fracture when at least 2 clinical criteria were present was 11% and the NPV 26%; however, specificity was 98% and PPV 96%. When at least 3 clinical criteria were present, the sensitivity and NPV were very low (6% and 25%), but both the specificity and PPV were 100%.

Of the 8 criteria included in the multivariate analysis, only 4 were significant predictors of nasal fracture (EPI, ECH, INF, and ASI). All 4 were included in the final logistic regression model, represented by the following formula: “*y* = 0.03 + 0.47EPI + 0.27ECH + 0.36INF + 0.26ASI” (Table 4). Of the 4 criteria, ASI had a maximum OR value (infinite) and the minimum 95% CI value (zero).

## DISCUSSION

Performance of clinical criteria for nasal fracture diagnosis was measured using sensitivity, specificity, PPV, and NPV. The sensitivity of a test is defined as the proportion of those with the disease who have a positive result, specificity is the proportion of those with no disease that have a negative result, PPV is the proportion of those with a positive test who actually have the disease, and NPV is the proportion of those with a negative test who do not have disease. Sensitivity and specificity are important measures of the diagnostic accuracy of a test but cannot be used to estimate the probability of disease among individual patients. Positive predictive value and NPV provide estimates of probability of disease, but both parameters are dependent on the prevalence of disease and vary accordingly. While sensitivity and specificity are important measures of the diagnostic accuracy of a test, they are not of practical use in the clinical setting, that is, to assist clinicians in estimating the probability of disease. For this purpose, PPV has greater utility and is more appropriate.^10^

Results of the current study are supported by previous research that suggests that clinical diagnosis of nasal fracture is approprtiate.^5^^,^^6^ Formerly, the presence of epistaxis after nasal trauma has been shown to be associated with a significant increased risk of external nasal deformity^11^; however, the current study is the first to examine the importance of other clinical criterion and their combinations and, for this reason, should be instructive in the clinical setting. The sensitivity and NPV for any single clinical criterion were very low (34% and 32%, respectively). Sensitivity and NPV were lower when 2 (11% and 26%, respectively) or 3 clinical criteria (6% and 25%, respectively) were present at the same time. Nevertheless, for the specificity and PPV were larger. Thus, 8% of patients with at least 1 clinical criterion present did not have nasal fracture; 92% of the patients without nasal fractures did not have any clinical criterion (specificity of 92%). Of patients with at least 1 clinical criterion, 94% had a nasal fracture (PPV of 94%). When at least 2 clinical criteria were present, only 2% of patients did not have nasal fracture although at least 2 clinical criteria will be present or 98% of the patients without nasal fractures will not have at least 2 clinical criteria at the same time (98% of specificity), and 96% of patients with at least 2 clinical criteria at the same time will have nasal fracture (PPV). When 3 clinical criteria are present, 100% of patients without nasal fractures did not have 3 clinical criteria (100% specificity) and 100% of patients with at least 3 clinical criteria did have a nasal fracture (100% PPV). Thus, when 3 or more clinical criteria are present, this study offers evidence that will facilitate an accurate diagnosis of nasal fracture in the emergency department only on the basis of clinical criteria. This could reduce the high cost associated to the use of radiographs or CT scans since they would not be necessary.

There is consensus that PPV has the highest value for clinicians in estimating the probability of disease^10^; thus, this measure should have the greatest clinical utility. Analyzing the results in a similar manner, the clinical criteria “epistaxis” is associated with a statistically significant increase in external nasal deformity and therefore a probably fracture.^11^ The high PPV for this clinical criterion indicates a high probability of real nasal fracture. For example, when at least only 1 clinical criterion is present, this probability will be 94%, and when at least 2 clinical criteria are present at the same time, this probability will be 96%, and finally, when at least 3 clinical criteria are present at the same time, this probability will be 100%. On the contrary, there are patients without epistaxis following nasal trauma with external nasal deformity; hence, they could have nasal fracture and still need to be referred to the fractured nose clinic.^11^ The low NPV of these results indicates that when our clinical criteria are absent, the clinicians cannot rule out the nasal fracture. Nevertheless, on the basis of the logistic regression formula calculated in the present study, “*y* = 0.03 + 0.47EPI + 0.27ECH + 0.36INF + 0.26ASI,” we can see that although it is possible, it is very difficult to have a nasal fracture (*y*) with no clinical criteria associated. This probability is given by the previous constant term (0.03). Based on the 220 patients evaluated, just 6 (2.73%) had a nasal fracture without associated clinical criteria.

Acute septal injury was the single clinical criterion with the maximum possible values for the specificity (100%), PPV (100%), and OR (infinite). Thus, if ASI is present, there always will be a nasal fracture associated. However, of all the clinical criteria considered in the analysis, ASI is the most difficult for a general physician or emergency physician to assess because a bright light and a nasal speculum are necessary for an accurate nasal septum inspection.

### Limitations of the study

This study had several limitations to be considered:

1. The sample of the study was small (220 patients).

2. The nasal trauma patients included in the current investigation consisted solely of those admitted to a maxillofacial department of a hospital for occupational injuries.

3. The strict inclusion criteria may have limited the number of patients eligible for the study.

4. In spite of its importance for nasal fracture diagnosis, palpation of the nasal bones was not included as a criterion for the analysis because when the nose is inflamed, it is difficult even for a specialist physician to assess the nasal bones crepitation.

## CONCLUSION

In the majority of cases, clinical criteria are useful for the diagnosis of nasal fractures; however, when criteria are absent, it is not possible to rule out nasal fracture, although this possibility is remote (2.73%).

## Acknowledgments

We thank Patricio Andrades, MD, for providing access to the database of the Maxillofacial Surgery Department at the “Hospital del Trabajador” (Chile), a hospital for occupational injuries.

## Figures and Tables

**Table 1:** Demographic and injury characteristics (N = 220)

Mean age, y	36 ± 25
Males (%)	157 (71%)
Females (%)	63 (29%)
Males/females	2.49/1
History of actual illness	29 (13%)
Previous nasal trauma (%)	28 (13%)
Traumatic mechanism (%)	
Motor vehicle collision	78 (35%)
Traumatism with a blunt instrument	45 (20%)
Fall accident	37 (17%)
Assault	20 (9%)
Unknown	41 (19%)
Nasal fractures (%)	167 (76%)

**Table 2:** Frequencies distribution and statistical measures of the performance of each clinical criterion

Clinical criterion	Percentage (N)	Sensitivity, %	Specificity, %	PPV, %	NPV, %
Epistaxis	54 (119)	69	94	97	50
Ecchymosis	20 (44)	26	98	98	30
Nasal wound	42 (92)	46	70	83	29
Airway obstruction	15 (33)	19	96	94	27
Inflammation	45 (98)	56	92	96	40
Lateral deviation	15 (32)	18	96	94	27
Irregular nasal dorsum	24 (52)	29	92	92	29
Acute septal injury	8 (17)	10	100	100	26
Mean	28	34	92	94	32

**Table 3:** Grouped clinical criteria performance

Clinical Criteria	Prevalence, % (n)	Sensitivity, %	Specificity, %	PPV, %	NPV, %
EPI + ECH	14 (31)	19	100	100	28
EPI + NW	23 (50)	30	100	100	31
EPI + AO	12 (26)	10	100	100	27
EPI + INF	16 (35)	21	100	100	29
EPI + LD	11 (24)	14	98	96	27
EPI + IND	17 (37)	22	98	97	28
EPI + ASI	7 (15)	9	100	100	26
INF + ECH	14 (31)	18	98	97	28
INF + NW	22 (48)	26	92	92	28
INF + AO	10 (22)	12	96	91	26
INF + LD	7 (16)	10	100	100	26
INF + IND	14 (31)	17	96	94	27
INF + ACI	5 (6)	6	100	100	25
NW + ECH	10 (21)	12	98	95	26
NW + AO	7 (15)	8	96	87	25
NW + ND	5 (11)	7	100	100	25
NW + IND	13 (29)	16	96	93	27
NW + ASI	4 (8)	5	100	100	25
IND + ECH	8 (17)	10	98	94	26
IND + AO	8 (18)	10	96	89	25
IND + LD	6 (14)	7	96	86	25
IND + ASI	3 (6)	4	100	100	25
ECH + AO	5 (10)	5	98	90	25
ECH + ND	3 (6)	4	100	100	25
ECH + ASI	2 (4)	2	100	100	25
AO + ND	5 (11)	7	100	100	25
AO + ASI	2 (5)	3	100	100	25
ND + ASI	2 (5)	3	100	100	25
**Mean**	**9**	**11**	**98**	**96**	**26**
**Mean for 3 CC**	**4**	**6**	**100**	**100**	**25**

ASI indicates acute septal injury; AO, airway obstruction; ECH, periorbital and/or perinasal ecchymosis; EPI, epistaxis; IND, irregular nasal dorsum; INF, nasal inflammation; LD, lateral deviation; NW, nasal wound or laceration.

**Table 4:** Logistic regression analysis

Variables	OR	95% CI
Epistaxis (EPI)	25.03	6.82-91.89^*a*^
Ecchymosis (ECH)	8.95	1.02-79.16^*a*^
Nasal Wound (NW)	2.35	0.95-5.79
Airway obstruction (AO)	1.00	0.12-8.66
Inflammation (INF)	5.97	1.80-19.85^*a*^
Lateral deviation (LD)	2.15	0.33-14.17
Irregular nasal dorsum (IND)	1.57	0.35-7.03
Acute septal injury (ASI)	∞	0.00^*a*^
Logistic regression formula: *y* = 0.03 + 0.47EPI + 0.27ECH + 0.36INF + 0.26ASI		

CI indicates confidence interval; OR, odds ratio.

^*a*^ These are the significant clinical criteria (1 value is not included in the 95% confidence interval) or independent variables. The 0 value for the confidence interval of the ASI means that if there is an ASI, there always will be a nasal fracture associated.
